# Supplementary material for: Exceptionally high work density of a ferroelectric dynamic organic crystal around room temperature
Source: Nat Commun. 2022 May 20;13:2823. doi: 10.1038/s41467-022-30541-y (PMC9123006; doi:10.1038/s41467-022-30541-y)
Supplement: Supplementary file 1 — Supporting Information [file 41467_2022_30541_MOESM1_ESM.pdf]

Supplementary Information

Exceptionally High Work Density of a Ferroelectric Dynamic Organic Crystal at Room Temperature

Supplementary figures

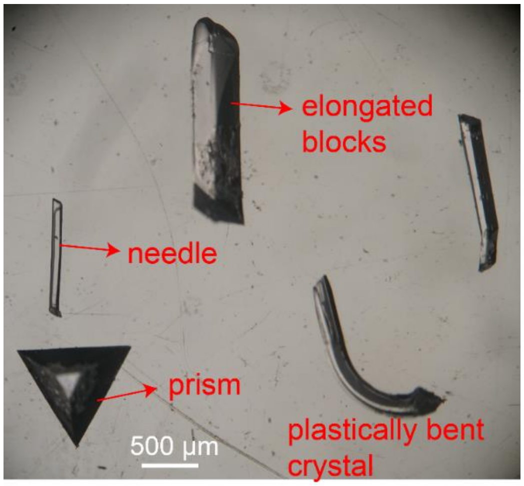

Supplementary Figure 1. Morphology and crystal habits of various guanidinium nitrate crystals at 285 K.

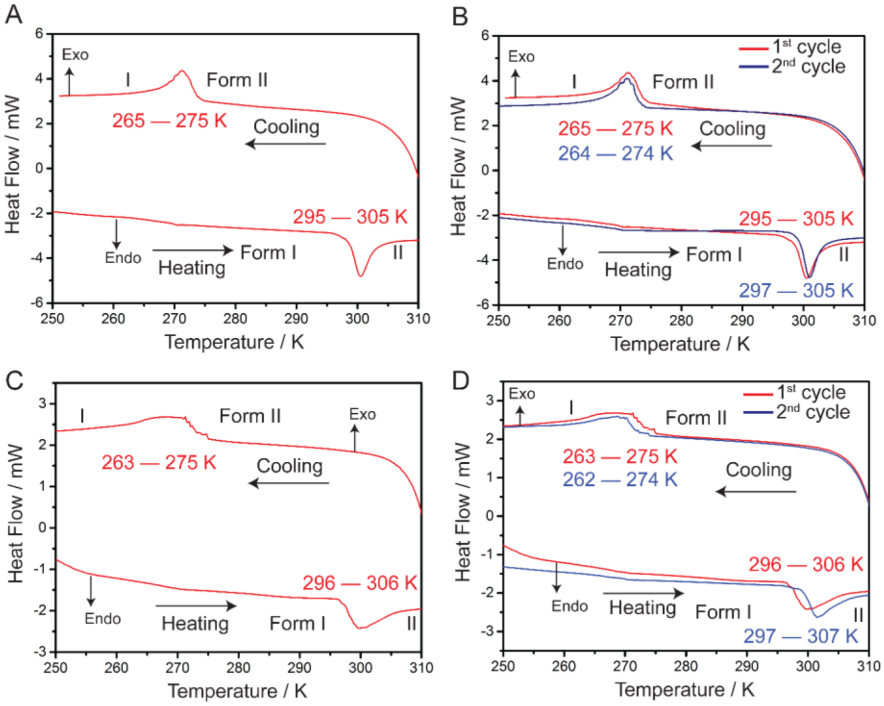

Supplementary Figure 2. Differential Scanning Calorimetry (DSC) of as-obtained (A, B) and lightly ground (C, D) crystals of form I of guanidinium nitrate (GN). (A) DSC profile recorded by heating and cooling of crystals over the temperature of phase transition. (B) DSC profile recorded over two consecutive thermal cycles. Note the slight offset in the range of transition temperatures between the consecutive cycles. (C) DSC profile of lightly ground crystals of GN. Note that similar to other thermosalient transitions (for example, *J. Am. Chem. Soc*. 2013, 135, 12241), the peak intensity is significantly decreased compared to the nonground crystals. (D) Two thermal cycles in the DSC of lightly ground crystals. The heating and cooling rates in all experiments were 10 K min^{-1}.

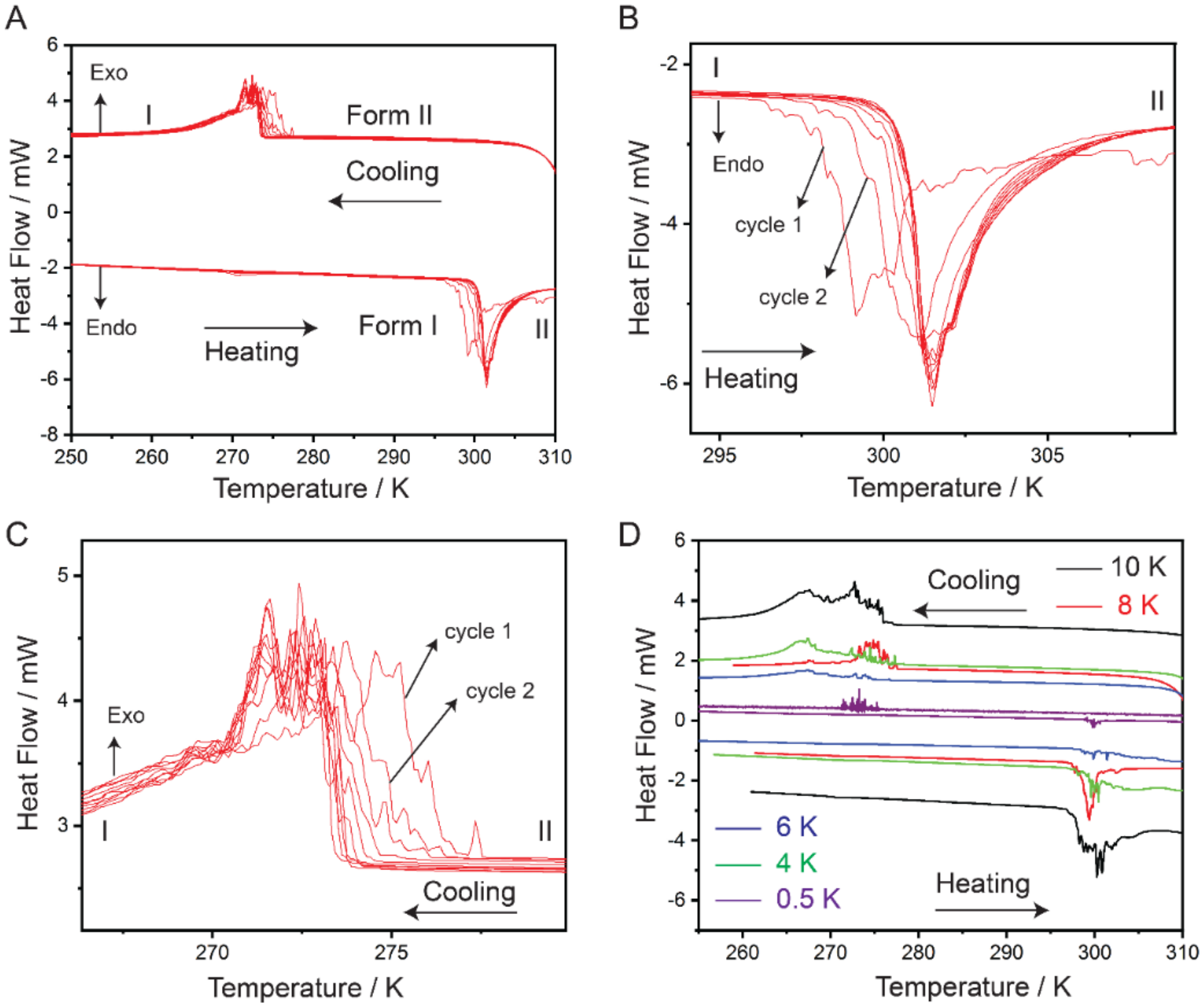

Supplementary Figure 3. (A, B, C) Differential Scanning Calorimetry (DSC) of single crystals of GN recorded over ten consecutive thermal cycles. (D) Effect of heating rate during heating and cooling cycles.

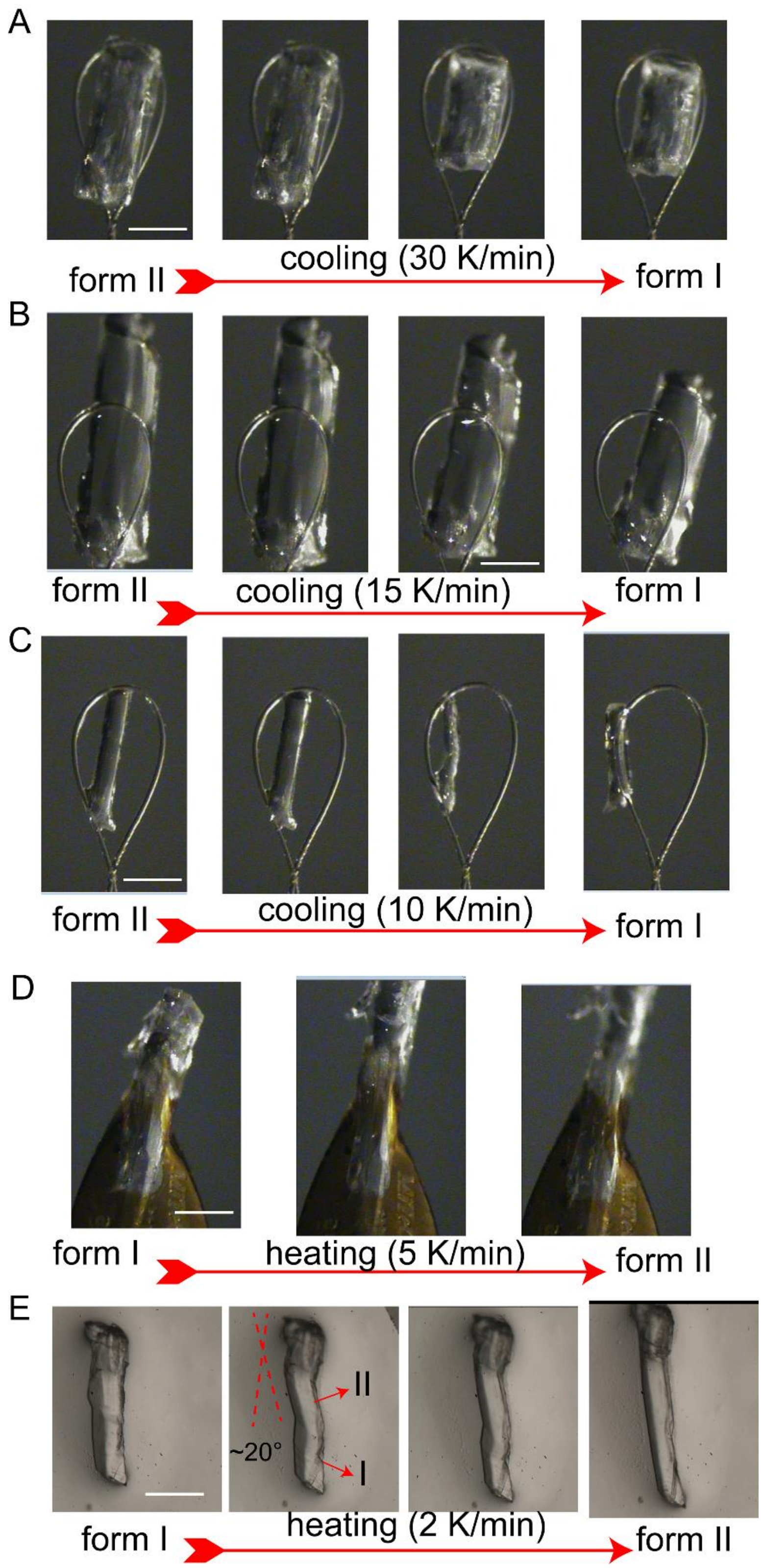

Supplementary Figure 4. Effect of heating and cooling rate during phase transition. Form II is converted to form I during cooling (A–C) and form I is converted to form II during heating (D–E). The length of the scale bars in panels A–D is 400 μm and in panel D it is 600 μm.

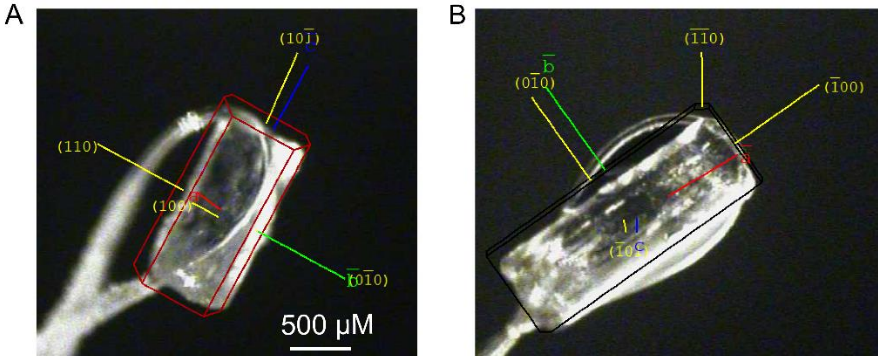

Supplementary Figure 5. Face indexing of form I (A) at 200 K and form II (B) at 305 K.

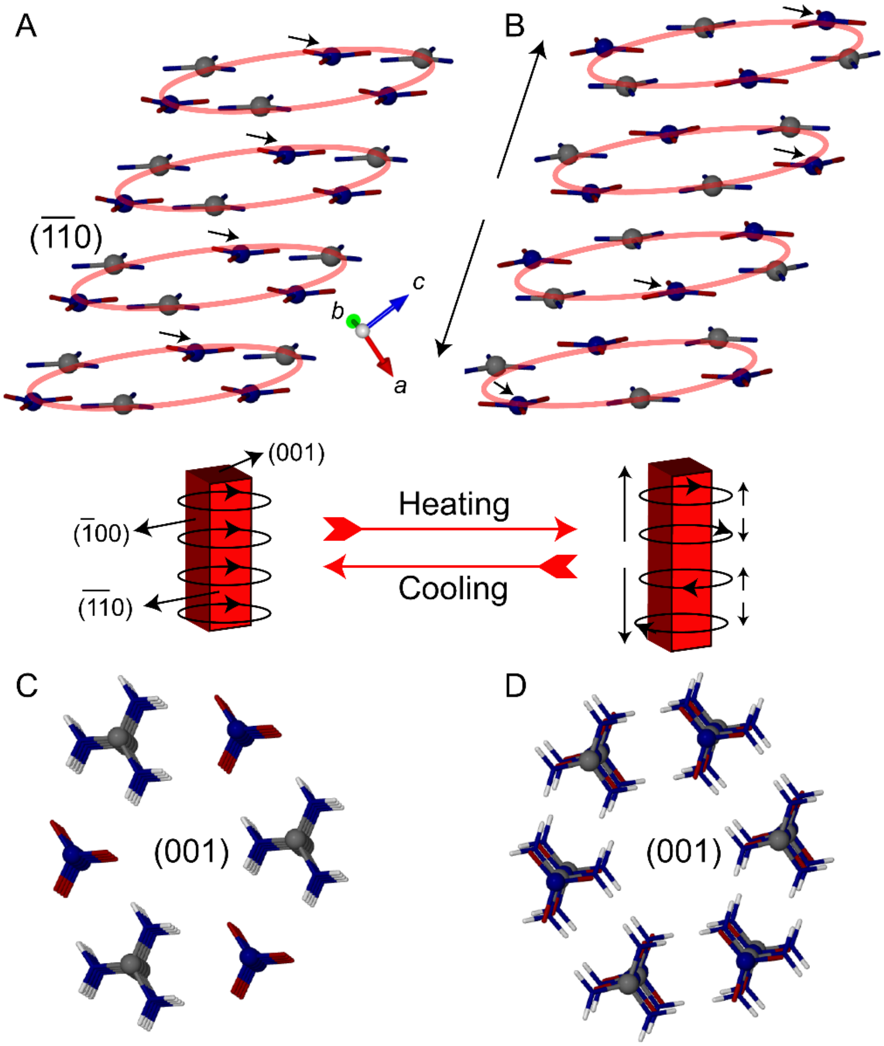

Supplementary Figure 6. Molecular structures and selected geometric features of the molecular packing and intermolecular interactions in forms I (A, C) and II (B, D) of guanidinium nitrate.

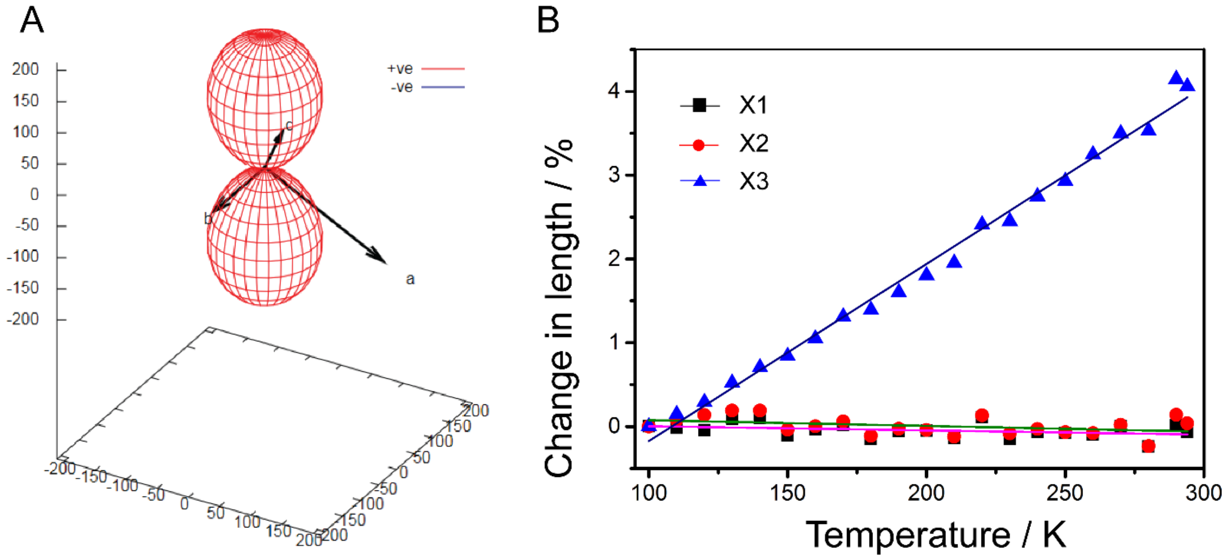

Supplementary Figure 7. Left: Expansivity tensor plot of form I guanidinium nitrate (monoclinic crystal system) calculated by using the software PASCal (Cliffe, M.; Goodwin, A. *J. Appl. Crystallogr*. 2012, 45, 1321–1329). Right: Percentage changes along the principal axes (X1, X2 and X3) lengths as a function of temperature.

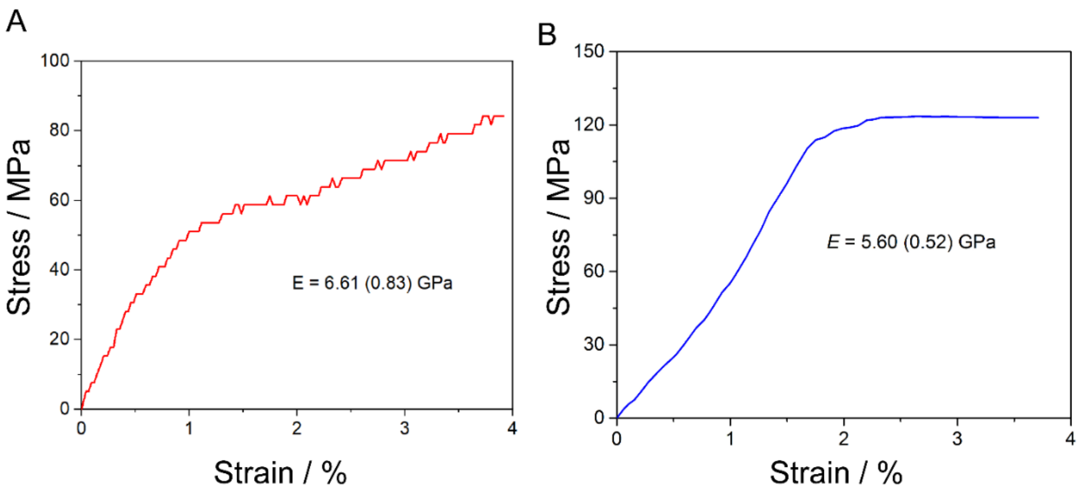

Supplementary Figure 8. Mechanical properties of a crystal of form I and II guanidinium nitrate. Panels A and B show the force-displacement and the stress-strain curves of form I and form II, respectively. The Young's moduli of form I and form II calculated from the stress-strain curve are 6.61 ± 0.83 GPa and 5.60 ± 0.52 GPa, respectively.

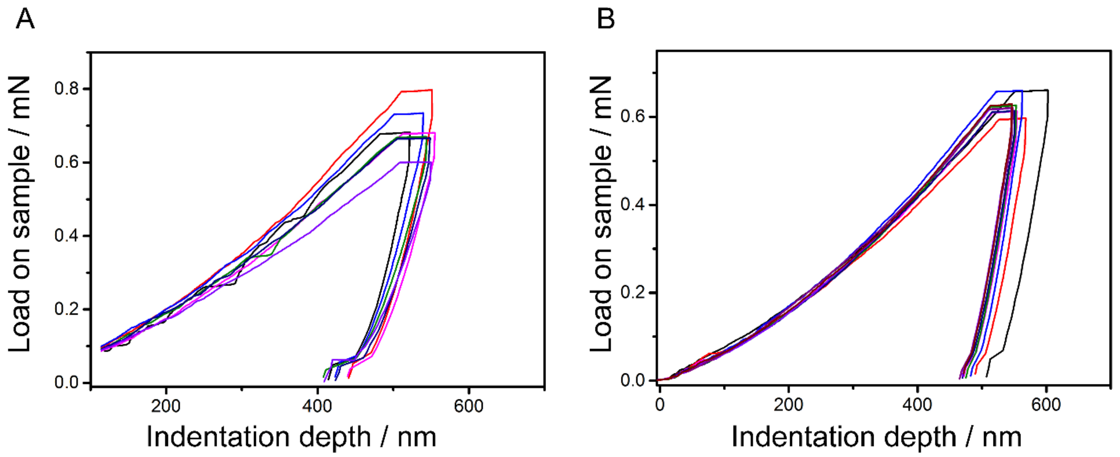

Supplementary Figure 9. Load-displacement curves of guanidinium nitrate crystals obtained by nanoindentation on the (100) face of form I (A) and form II (B).

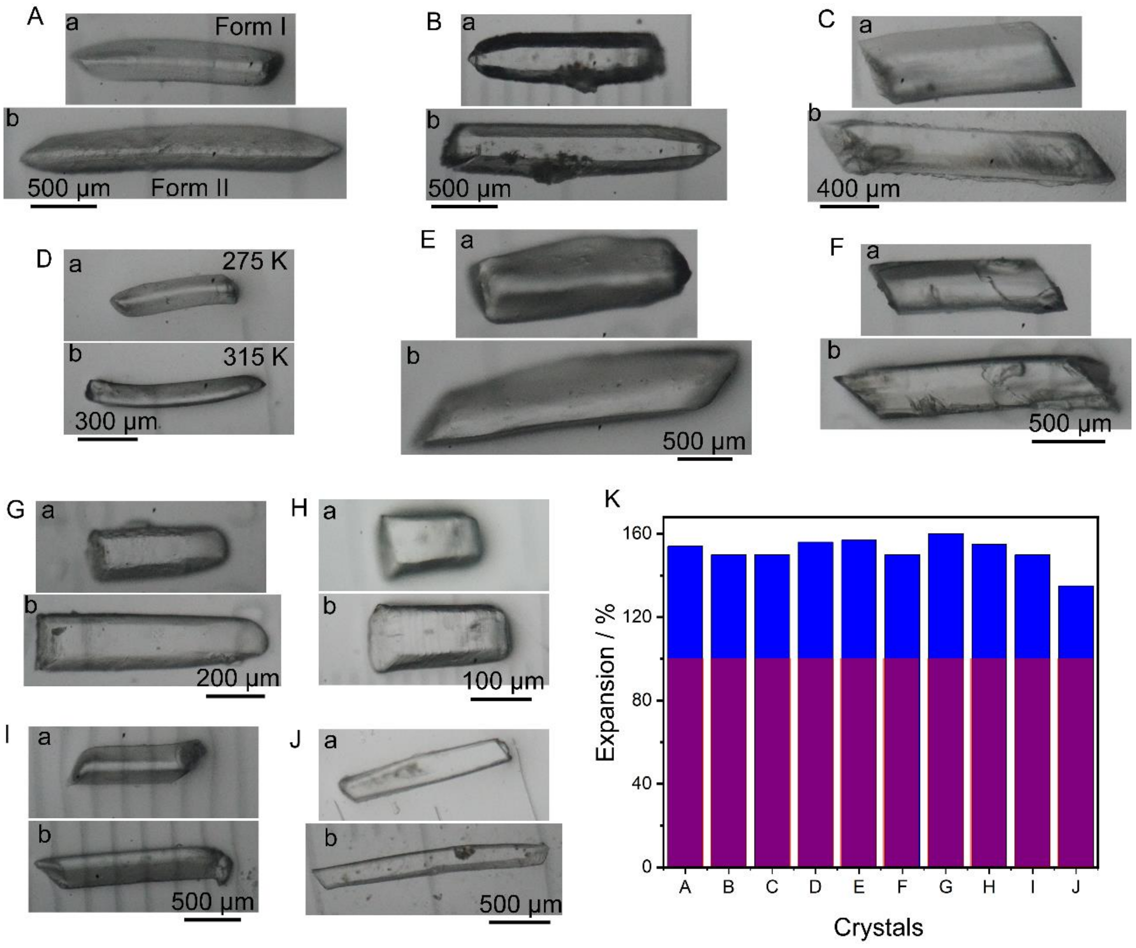

Supplementary Figure 10. Changes in size and shape of single crystals of form I guanidinium nitrate during heating.

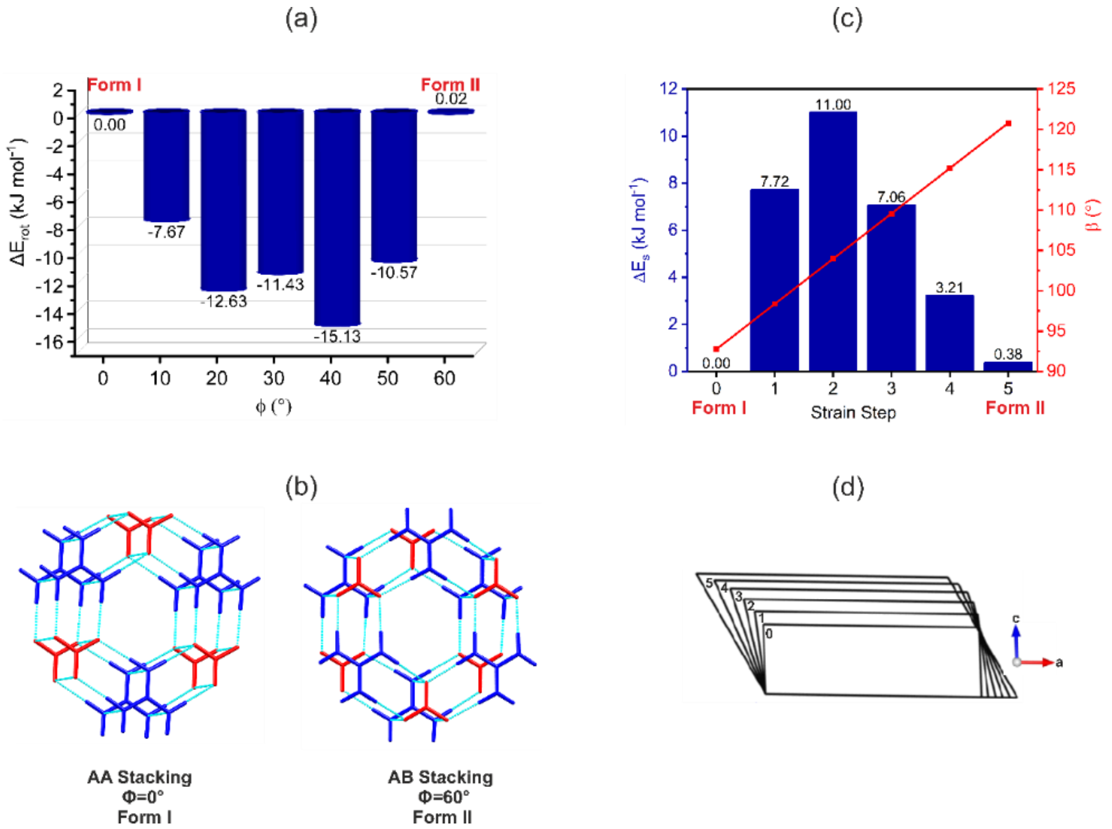

Supplementary Figure 11. (a) DFT-D estimate of the rotational energy profile for switching between the AA Form I stacking ($\phi = 0°$) to the AB Form II stacking ($\phi = 60°$) of the ions. (b) Snapshots of the relative orientations of the rings in the two phases. (c) DFT-D estimate of the relative strain energy for stepwise transformation of the equilibrium cell geometry of Form I (Strain Step 0) to the equilibrium cell geometry in Form II (Strain Step 5). The stepwise cell transformation is indicated in the plot by the linear increase in the β angle. At each strain step, the ions are allowed to relax at fixed volume. (d) Overlay of the cell geometries in the simulations shown in panel (c). The numbers shown correspond to the strain step.

Supplementary tables

Supplementary Table 1. Crystallographic data and refinement details of guanidinium nitrate structures refined from the same crystal at different temperatures

**Temperature / K**	**250 K**	**310 K**
**Polymorph**	**Form I**	**Form II**
Formula weight	122.10	122.10
Crystal system	Monoclinic	Monoclinic
Space group	*Cm*	*C*2
a / Å	10.925(3)	12.694(3)
b / Å	7.2724(15)	7.2829(19)
c / Å	3.6049(8)	7.2555(18)
α / °	90.00	90.00
β / °	93.613(8)	120.828(9)
γ / °	90.00	90.00
Volume / Å^3	285.84(12)	576.0(3)
Z	2	4
Density / (g cm^{-3})	1.42	1.41
μ / mm^{-1}	0.134	0.133
F_{000}	128.0	256.0
h_{min}, h_{max}	-13, 13	-14, 14
k_{min}, k_{max}	-8, 8	-8, 8
l_{min}, l_{max}	-4, 4	-8, 8
No. of measured reflections	1046	3497
No. of unique reflections	573	970
No. of reflections used	520	615
R_{all}, R_{obs}	0.0514, 0.0465	0.1162, 0.0720
$wR_{2,all}$, $wR_{2,obs}$	0.1167, 0.1134	0.1939, 0.1730
$\Delta\rho_{min,max}$ / (e Å^{-3})	-0.163, 0.171	-0.334, 0.307
GooF	1.159	1.221
CCDC No.	2123337	2123338

Supplementary Table 2. Temperature-dependent change of the three axes of the crystallographic axes of a form I guanidinium nitrate

T / K	*a* / Å	sd (*a*)[a]	*b* / Å	sd (*b*)[a]	*c* / Å	sd (*c*)[a]
100	10.865	0.004	7.282	0.002	3.514	0.001
110	10.863	0.003	7.293	0.002	3.516	0.001
120	10.864	0.003	7.292	0.002	3.523	0.001
130	10.882	0.003	7.296	0.002	3.53	0.001
140	10.887	0.003	7.296	0.002	3.536	0.001
150	10.87	0.003	7.279	0.001	3.539	0.001
160	10.883	0.003	7.282	0.002	3.545	0.001
170	10.896	0.003	7.283	0.002	3.553	0.001
180	10.88	0.003	7.274	0.002	3.554	0.001
190	10.892	0.003	7.28	0.002	3.561	0.001
200	10.891	0.003	7.279	0.002	3.567	0.001
210	10.92	0.003	7.273	0.002	3.572	0.001
220	10.909	0.003	7.292	0.002	3.587	0.001
230	10.919	0.003	7.271	0.002	3.586	0.001
240	10.914	0.003	7.277	0.002	3.595	0.001
250	10.921	0.003	7.277	0.003	3.602	0.001
260	10.93	0.004	7.275	0.002	3.611	0.001
270	10.907	0.005	7.282	0.003	3.621	0.001
280	10.953	0.004	7.265	0.002	3.621	0.001
290	10.939	0.005	7.283	0.003	3.644	0.002
294	10.939	0.003	7.277	0.002	3.639	0.001

[a]Standard deviation.

Supplementary Table 3. Selected coefficients of uniaxial thermal expansion (TE) and their volumetric thermal expansion (VTE) reported

Compound or acronym in the original publication	Temp. range (K)	Uniaxial TE α_x (10^{-6} K^{-1})	VTE α_V (10^{-6} K^{-1})	Reference
(phenylazophenyl)palladium(II)(hexafluoroacetylacetonate)	220-350<br><br>100-270	260.4, 39.4, -79.9.<br>124.0, 105.4, 114.9	247.8 (α form)<br>255.5 (β form)	1
18-crown-6-nitromethane	180-273	X1−129(15)<br>X2−144(14)<br>X3−282(16)	311	2
4-aminobenzonitrile and 4-(dimethylamino)benzonitrile in 1:2	100-300	X1−24.3<br>X2−90.9<br>X3−105	222	3
1,2,3,4-cyclobutane-tetracarboxylic acid and 4,4′-bipyridylethylene	120-298	X1−4(5)<br>X2−25(4)<br>X3−147(8)	183	4
4-phenylazopyridine 4,6-diCl and 4,6-dichlororesorcinol in 2:1 ratio	290-260	X1−116<br>X2−29<br>X3−316	229	5
FMOF1	90-295	230	300	6
4-aminobenzonitrile (ABN)	180-300	X1−24.8<br>X2−61.3<br>X3−138.2	225.1	7
triphenylethenyl gold isocyanide complex	123-298	X1−285.2<br>X2−22.1<br>X3−579.1	315.3	8
1D coordination polymer of lead(II)	298-398	X1−53.7<br>X2−63.2<br>X3−189.9	200.7	9
$[Pb(SCN)_2(2F\text{-}spy)_2]$	173- 393	X1−6.3<br>X2−35.2<br>X3−93.4	136.4	10
$[Zn(benzoate)(25F\text{-}spy)_2]$	303-413	X1−54.46<br>X2−106.42<br>X3−151.69	204.26	11
$[Cu_2(benzoate)_4(L)_2]$,<br>L = 4-styrylpyridine (4spy)	273-473	X1−13.9<br>X2−56.0<br>X3−166.3	241.8	12
2′-fluoro-4-styryl-pyridine (2F-4spy)	273-473	X1−21.8<br>X2−38.3<br>X3−167.7	233.1	12
3′-fluoro-4-styrylpyridine	273-473	X1−13.8<br>X2−64.5<br>X3−228.3	285.6	12
MCF-82	112-300	X1−61<br>X2−482<br>X3−218	319	13

(*S*,*S*)-octa-3,5-diyn-2,7-diol	225-330	156<α_a>516<br>-32<α_b>-85<br>-48<α_a>-204		14
Olefin-I and several derivatives	190-250	X1−2<br>X2−68<br>X3−128	194	15
Form I of compound IMACET	298-373	α_a 225.9<br>α_b 238.8<br>α_c −290.0	181.2	16
4PAzP	260-290	X1−116<br>X2−29<br>X3−316	328	17
4,4′-AP and 4,6-diX-res and other derivatives	190-290	X1−2<br>X2−17<br>X3−164	185	18
{[FeTp(CN)$_3$]$_2$Co(Bib)$_2$}·5 H_2O	180-240	X1−85<br>X2−278<br>X3−1089	1498	19
ABN·2DMABN	100-300	α_{X1} 24.3<br>α_{X2} 90.9<br>α_{X3} 105.0	222	20
DAN-CA	85-385	27< αa < 33<br>56 < αb < 78<br>35 < αc < 58		21
24DNAN, the β-form	100-261	X1−31(6)<br>X2−15(7)<br>X3−262(20)	216	22
{[Zn(BTC)(HBPP)]·H_2O}$_n$ (**1**·H_2O)	260-100	a, b and c are −6.0(1), 53.0(7) and −7.9(5)	39	23
(Himd)$_2$[CuCl$_4$]		$\alpha_{a'}$ = −38 × 10 $^{-6}$ K^{-1}, $\alpha_{b'}$ = 5 68 × 10^{-6} K^{-1}, and $\alpha_{c'}$ = −18 4 × 10^{-6} K^{-1}	346	24
IMD–HBC	100-360	X1−115<br>X2−18<br>X3−210	110	25
Trianglimine form II	Cooling	X1−17(1)<br>X2−28(1)<br>X3−145(5)	161	26
Trianglimine form III	Cooling	X1−5(2)<br>X2−59(1)<br>X3−98(2)	151	26
Guanidinium nitrate	100-294	X1−4.98<br>X2−6.81<br>X3−211.46	210.79	Current work

Supplementary Table 4. Reported crystals and their axial thermal expansions in percentage

Crystal	Maximum Axial Thermal Expansion	References
[Ni(II)(ethylenediamine)$_3$](oxalate anion) complex	5%	*Nat. Chem*. 2014, 6, 1079-1083.
Organic–inorganic hybrid Cu(II) complex, bis(imidazolium) tetrachlorocuprate, (Himd)$_2$[CuCl$_4$]	~10%	*Nat. Commun*. 2019, 10, 4805.
Cobalt(II) complex with a *n*-butyl group in its ligand, [Co(NO$_3$)$_2$(L)]	6 – 7%	*Nat. Commun*. 2015, 6, 8810.
Ag$_3$[Co(CN)$_6$]	~6%	*Science* 2008, 319, 794-797.
Pyridine–ICl and pyridine–IBr complexes	~2.7% and ~2.9%	*CrystEngComm* 2014, 16, 237-243.
IMACET I-II (irreversible)	12.3%	*Sci. Rep*. 2016, 6, 29610.
Pentacene and rubrene	~2.6% and ~2.3%	*J. Phys. Chem. Lett*. 2012, 3, 3325-3329.
α-(phenylazophenyl)palladium hexafluoroacetylacetonate	~4.6%	*Nat. Commun*. 2014, 5, 4811.
7,7,8,8-tetracyanoquinodimethane-*p*-bis(8-hydroxyquinolinato)copper(II) (not reversible)	100%	*J. Am. Chem. Soc*. 2014, 136, 590-593.
2,7-di([1,1′-biphenyl]-4-yl)-fluorenone	~10%	*Nat. Commun*. 2019, 10, 4573.
Guanidinium nitrate (reversible)	51.8%	This work
naphthalenediimide (NDI) systems (crystal shrinks upon cooling)	-10 % (shrink)	*J. Am. Chem. Soc.* 2021, 143, 5951–5957
[Ni(II)(en)$_3$](ox) complex (1)	-5 % (shrink)	*Nat. Chem*. 2014, 6, 1079–1083.
TIPS-pentacene	10 %	*Nat. Commun*. 2018, 9, 278.
N-[[4-*p*-dimethylaminophenylazo]benzoyl]-1-phenylethylamine [trans-(*S*)-1]	4.5% (along width)	*Nat. Commun*. 2018, 9, 538.
[Co(II)(en)$_3$](ox) (en = ethylenediamine) (crystal shrinks upon cooling)	-4.5% (shrink)	*Angew. Chem. Int. Ed*. 2017, 56, 717 –721.
[(H$_4$BPTC)(azpy)$_2$]$_n$ (crystal shrinks upon cooling)	-4% (shrink)	*Angew. Chem. Int. Ed*. 2016, 128, 14848–14852

Supplementary Table 5. Measured properties of guanidinium nitrate form I crystals

Crystal	Length (mm)	Width (mm)	Thickness (mm)	Mass (mg)	Force (mN)	Force Density (N m^{-3})	Stroke (m)	Work (J)	Work density (J m^{-3})
1	0.7	0.3	0.1	2.1E-02	1.34	6.37E+07	3.50E-04	1.05E-07	2.23E+04
2	1	0.5	0.3	9.5E-02	2.34	1.56E+07	5.00E-04	2.50E-07	7.80E+03
3	1.5	0.8	0.8	1.7E-01	21.6	2.25E+07	7.50E-04	6.00E-07	1.69E+04
4	1.6	0.7	0.4	1.5E-01	13.4	2.98E+07	8.00E-04	5.60E-07	2.39E+04
5	1.9	0.6	0.7	2.0E-01	35.5	3.81E+07	9.50E-04	6.65E-07	3.62E+04
6	1.9	0.6	0.5	1.8E-01	48.8	8.56E+07	9.50E-04	5.70E-07	8.13E+04

Supplementary Table 6. Frequency dependent conductance (G)-voltage measureents on single crystal before (form I) and after phase transition (form II)

	Form I		Form II	
Frequency (kHz)	Capacitance (nF)	Conductance (ms)	Capacitance (nF)	Conductance (ms)
200	6.2	2.2	55	74
100	29	4	103	79
50	118	14.4	167	80
10	137	80	2000	173
1	110	63.5	25300	528

Supplementary references

1. Panda, M. K. et al. Colossal positive and negative thermal expansion and thermosalient effect in a pentamorphic organometallic martensite. *Nat. Commun*. **5**, 4811 (2014).

2. Engel, E. R.; Smith, V. J.; Bezuidenhout, C. X. & Barbour, L. J. Uniaxial negative thermal expansion facilitated by weak host–guest Interactions. *Chem. Commun*. **50**, 4238–4241 (2014).

3. Alimi, L. O.; Lama, P.; Smith, V. J. & Barbour, L. J. Large volumetric thermal expansion of a novel organic cocrystal over a wide temperature range, *CrystEngComm* **20**, 631–635 (2018).

4. Bhattacharya, S. & Saha, B. K. Interaction dependence and similarity in thermal expansion of a dimorphic 1D hydrogen-bonded organic complex. *Cryst. Growth Des*. *13*, 3299–3302 (2013).

5. Hutchins, K. M.; Groeneman, R. H.; Reinheimer, E. W.; Swenson, D. C. & MacGillivray, L. R. Achieving dynamic behaviour and thermal expansion in the organic solid state via co-crystallization. *Chem. Sci*. **6**, 4717–4722 (2015).

6. Yang, C.; Wang, X. & Omary, M. A. Crystallographic observation of dynamic gas adsorption sites and thermal expansion in a breathable fluorous metal–organic framework. *Angew. Chem. Int. Ed*. **48**, 2500–2505 (2009).

7. Alimi, L. O., van Heerden, D. P., Lama, P., Smith, V. J. & Barbour, L. J. Reversible thermosalience of 4-aminobenzonitrile. *Chem. Commun*. 54, 6208–6211 (2018).

8. Seki, T., Mashimo, T., Ito, H. Anisotropic strain release in a thermosalient crystal: correlation between the microscopic orientation of molecular rearrangements and the macroscopic mechanical motion. *Chem. Sci*. 10, 4185– 4191 (2019).

9. Rath, B. B., Gallo, G., Dinnebier, R. E., Vittal, J. J. Reversible Thermosalience in a One-Dimensional Coordination Polymer Preceded by Anisotropic Thermal Expansion and the Shape Memory Effect. *J. Am. Chem. Soc*. 143, 2088– 2096 (2021).

10. Rath, B. B., Gupta, M., Vittal, J. J. Multistimuli-Responsive Dynamic Effects in a One-Dimensional Coordination Polymer. *Chem. Mater*. 2022, 34, 178–185.

11. Bolla, G. *et al.* Multifunctional Properties of a Zn(II) Coordination Complex. *Cryst. Growth Des.* 2021, 21, 3401–3408.

12. Yadava, K. et al. Extraordinary anisotropic thermal expansion in photosalient crystals. *IUCrJ* 7, 83–89 (2020).

13. Zhou, H. L., Zhang, Y. B., Zhang, J. P. & Chen, X. M. Supramolecular-jack-like guest in ultramicroporous crystal for exceptional thermal expansion behaviour. *Nat. Commun*. 6, 6917 (2015).

14. Das, D., Jacobs, T. & Barbour, L. J. Exceptionally large positive and negative anisotropic thermal expansion of an organic crystalline material. *Nature Mater*. 9, 36–39 (2010).

15. Ding, X., Zahid, E., Unruh, D. K., & Hutchins, K. M. Differences in thermal expansion and motion ability for herringbone and face-to-face π-stacked solids. *IUCrJ*, 9, 31-42 (2022).

16. Panda, M. K. et al. Strong and Anomalous Thermal Expansion Precedes the Thermosalient Effect in Dynamic Molecular Crystals. *Sci. Rep*. 6, 29610 (2016).

17. Hutchins, K. M., Groeneman, R. H., Reinheimer, E. W., Swenson, D. C. & MacGillivray, L. R. Achieving dynamic behaviour and thermal expansion in the organic solid state via co-crystallization. *Chem. Sci*. 6, 4717–4722 (2015).

18. Hutchins, K. M. *et al.*, Thermal Expansion Properties of Three Isostructural Co-Crystals Composed of Isosteric Components: Interplay between Halogen and Hydrogen Bonds. *CrystEngComm*, 18, 8354– 8357 (2016).
19. Hu, J. X. *et al.,* A Material Showing Colossal Positive and Negative Volumetric Thermal Expansion with Hysteretic Magnetic Transition. Angew. Chem., Int. Ed. 56, 13052– 13055 (2017).
20. Alimi, L. O., Lama, P., Smith, V. J. & Barbour, L. J. Large Volumetric Thermal Expansion of a Novel Organic Cocrystal over a Wide Temperature Range. CrystEngComm 20, 631– 635 (2018).
21. Behera, R. K., Goud, N. R., Matzger, A. J., Brédas, J.-L. & Coropceanu, V. Electronic Properties of 1,5-Diaminonaphthalene:Tetrahalo-1,4-benzoquinone Donor-Acceptor Cocrystals. *J. Phys. Chem. C*, 121, 23633– 23641 (2017).
22. Takahashi, H. & Tamura, R. Low temperature phase transition induced biaxial negative thermal expansion of 2,4-dinitroanisole. *CrystEngComm*, 17, 8888– 8896 (2015).
23. Lama, P., Alimi, L. O., Das, R. K. & Barbour, L. J. Hydration-dependent anomalous thermal expansion behaviour in a coordination polymer. *Chem. Commun*., 52, 3231– 3234 (2016).
24. Yao, Z.-S. et al. Giant anisotropic thermal expansion actuated by thermodynamically assisted reorientation of imidazoliums in a single crystal. *Nat. Commun.* **10**, 4805 (2019).
25. Dwivedi, B., Shrivastava, A., Negi, L. & Das, D. Colossal Positive and Negative Axial Thermal Expansion Induced by Scissor-like Motion of 2D Hydrogen Bonded Network in an Organic Salt. Cryst. Growth Des. 19, 2519– 2524 (2019).
26. Janiak, A., Esterhuysen, C. & Barbour, L. J. A thermo-responsive structural switch and colossal anisotropic thermal expansion in a chiral organic solid. *Chem. Commun*. 54, 3727–3730 (2018).
