# Supplementary material for: Exceptionally high work density of a ferroelectric dynamic organic crystal around room temperature
Source: Nat Commun. 2022 May 20;13:2823. doi: 10.1038/s41467-022-30541-y (PMC9123006; doi:10.1038/s41467-022-30541-y)
Supplement: Supplementary file 3 — Description to Additional Supplementary Information [file 41467_2022_30541_MOESM3_ESM.pdf]

Legends to the Supplementary Movies

Supplementary Movie 1.

Expansion of GN single crystal during phase transition from form I to form II upon heating.

Supplementary Movie 2.

Contraction of GN single crystal during phase transition from form II to form I upon cooling.

Supplementary Movie 3.

Effect of temperature heating rate on the crystal morphology and the phase boundaries during phase transition.

Supplementary Movie 4.

Snakelike ‘slithering’ motion of long GN crystals during heating
